# Corneal parameters in diabetics versus non-diabetics and correlation with various blood sugar parameters

**DOI:** 10.22336/rjo.2024.24

**Published:** 2024

**Authors:** Shailaja Pandey, Archana Singh, Harikrishnan Vannadil, Mohini Agrawal

**Affiliations:** * Department of Ophthalmology, INHS Jeevanti, Goa, India; ** Department of Ophthalmology, INHS Asvini, Mumbai, India; *** Department of Ophthalmology, Eyeris Eye Care Hospital, Hyderabad, India; **** Department of Ophthalmology, Military Hospital, Jalandhar, India

**Keywords:** diabetes mellitus, endothelium, corneal parameters, endothelial counts, central corneal thickness, cell density

## Abstract

**Aim:** To compare corneal parameters in diabetics versus age-group-matched non-diabetics; also, to correlate these parameters with the duration of diabetes, glycated haemoglobin (HbA1c) levels, and severity levels of diabetic retinopathy (DR).

**Materials and methods:** A comparative study was conducted at a tertiary eye-care center from January 2020 to December 2020. Two-hundred patients (400 eyes) with type-2 diabetes (100) and age-sex-matched non-diabetics (100) were included. Corneal morphological parameters like central corneal thickness (CCT), endothelial cell density (ECD), coefficient of variance (CoV), hexagonality (6A), and average cell area were recorded by specular microscopy. These parameters were correlated with the duration of diabetes, severity of disease based upon fasting blood glucose levels, HbA1c, and grade of DR. Mean and standard deviation were calculated, and regular distribution of continuous data was tested using independent sample t-test and ANOVA.

**Results:** Mean ECD (2447.32 ± 269.89/mm2), 6A (45.03 ± 6.71%), and IOP (15.47 ± 2.02 mmHg) changed in diabetic cases and were significantly low in diabetics, whereas, mean average cell area (413 ± 50.19 mm2), standard deviation (167.05 ± 77.91), CCT (525.81 ± 36.69) and CoV (39.84 ± 15.59%), were significantly high in diabetics. Mean CCT had insignificant variation. Subgroup analysis within diabetics showed a statistically significant reduction of ECD, cell count, and 6A with increased duration of diabetes, poor glycaemic control, and raised HbA1c.

**Discussion:** The corneal endothelial analysis is vital in daily clinical practice and provides valuable evidence concerning the viability of corneal endothelium in various intraocular surgeries. Uncontrolled DM harms the cornea with 70% of diabetics resulting in complications like keratopathy. The study highlighted that the increased duration of diabetes raised HbA1c, and poor glycemic control negatively affected corneal morphology. Our study showed a definite reduction in ECD and 6A in diabetics compared to non-diabetics.

**Conclusion:** A definite reduction in the corneal endothelial counts, cell density, and hexagonality was found in type-2 diabetics compared to non-diabetics.

**Abbreviations:** DM = Diabetes Mellitus, CCT = central corneal thickness, ECC = endothelial cell counts, ECD = endothelial cell density, CoV = coefficient of variance, 6A = hexagonality, DR = Diabetic retinopathy, SD = Standard of deviation, IOP = Intraocular pressure

## Introduction

Diabetes Mellitus (DM) is a disorder characterized by employing a disturbance of carbohydrate fat and protein metabolism resulting in hyperglycemia, which causes pathological and functional changes affecting almost every organ in the human body [**1**,**2**]. Amongst the various ocular side-effects of DM, diabetic keratopathy, diabetic retinopathy (DR), diabetic cataractous eyes, and dry eye, the primary pathological change is basement membrane abnormality and microangiopathy [**3**-**6**]. Corneal endothelium shares the exact mesodermal origin as capillary endothelium. Hence, these are likely to share similar metabolic requirements. Thus, metabolic alteration in DM affects the corneal endothelium [**7**]. Based on this hypothesis, this study compared various corneal endothelial parameters, endothelial cell counts (ECC), density (ECD), standard of deviation (SD), coefficient of variance (CoV), hexagonality (6A), and central corneal thickness (CCT) in 100 diabetic subjects and 100 age-sex matched, non-diabetic subjects.

The corneal endothelial analysis is vital in daily clinical practice and provides valuable evidence concerning the viability of corneal endothelium in various intraocular surgeries [**8**-**10**]. Despite many studies on changes in corneal endothelial parameters in diabetics, the relationship is not clarified. Several studies have shown reduced ECD, increased CoV, and increased CCT, while others have reported no difference between diabetic and non-diabetic subjects [**11**-**14**]. The reasons for these contradictory results are attributed to various non-modifiable aspects such as race, ethnicity of subjects, and stage of diabetes.

Thus, we compared the corneal parameters between age- and sex-matched subjects with or without Type-2 DM. In addition, the stage of diabetes, HbA1C level, presence/absence of DR, and their effect on corneal morphology have also been studied. 

## Materials and methods

This observational study was conducted at the Department of Ophthalmology at a tertiary care hospital in India. The protocol followed the tenets of the Declaration of Helsinki and was approved by the institutional ethical committee. Written consent was obtained from each patient.

Type-2 diabetics and controls, 30 years or older, who attended ophthalmology OPD, were selected. Patients with active ocular inflammation, media opacity precluding fundus examination, a known case of glaucoma, a history of intraocular surgery, a history of contact lens use, or corneal diseases (keratoconus, dry eye, endothelial dystrophies) were excluded from the study.

DM was confirmed by medical history and HbA1C > 6.5%. All subjects had a complete ophthalmological examination using a standard protocol by a single ophthalmologist, including the staging of DR (based on ETDRS fundus photographs). A non-contact specular microscope analyzed CCT and corneal endothelial morphology (Tomey Specular EM-4000). The analyzed parameters were ECC, minimum and maximum cell size, ECD, average cell area, CoV in cell area, and 6A. An average of three measurements was used for each parameter of each eye. 

To assess serum HbA1C, all patients sampled venous blood on the same visit. Diabetic subjects were classified into subgroups according to fasting blood glucose level (< 126 mg/dl, 126-200 mg/dl, 201-400 mg/dl, and > 400 mg/dl), HbA1c levels (< 6.5%, 6.5-8%, 8-10% and > 10%) and presence or absence of DR.

*Statistical analysis*

Statistical analysis was performed using SPSS version 21.0 software. Categorical variables were shown by frequency and percentage in various tables and figures. Mean and SD were calculated, and the normal distribution of continuous data was tested using the independent sample t-test and ANOVA. p < 0.05 was statistically significant. 

## Results

Two hundred cases (400 eyes) were enrolled, out of which, 100 were diabetics, and 100 were observed in the non-diabetic group. Demographic characteristics of the study population had insignificant differences between the groups. The mean age of presentation was 48 ± 10 years. Hypertension was the only comorbid condition noted in both groups (25% and 21% respectively), which was not statistically significant. A maximum number of cases had a duration of diabetes up to 1 year, followed by those with more than 10 years (**Table 1**). Most of the diabetic patients had blood sugar levels of 126-200 mg/dl range (44%), and HbA1c level was noted between 6.5-8.0% in most of the diabetics (40%) (**Table 2**). Out of 200 eyes in the diabetic group, 172 (86%) did not have DR, whereas 22 eyes (11%) had non-proliferative DR and only 6 eyes (3%) had proliferative DR.

**Table 1:** Distribution of diabetic cases according to duration of illness

SN	Duration	No./% of cases
1.	≤ One year	27
2.	One-Two years	11
3.	Two-Five years	18
4.	Five-Ten years	22
5.	> Ten years	22

**Table 2:** Distribution of diabetic cases according to levels of fasting blood sugar and HbA1c levels

SN	Variable	No./% of cases
1.	Fasting blood sugar levels (mg/dl)	
	≤ 126 mg/dl	25
	126-200 mg/dl	44
	201-400 mg/dl	28
	> 400 mg/dl	3
2.	HbA1c level (%)	
	< 6.5	32
	6.5-8.0	40
	8.1-10.0	19
	> 10.0	9
3.	Urinary albumin creatinine ratio (UACR)	
	Normal (< 30)	95
	Microalbuminuria (30-300)	5
	Macroalbuminuria (> 300)	0

The best corrected visual acuity (BCVA) between the diabetic and non-diabetic groups showed a statistically significant difference (p < 0.001) with a BCVA between 6/6-6/9 in 92.5% of non-diabetic subjects, whereas it was < 6/9-618 in 78% of diabetics.

Regarding corneal morphology, the mean ECC, ECD, and 6A were considerably lower in cases than controls. In contrast, the mean average cell area, SD, and CoV were substantially higher in cases than controls (**Table 3**). The mean CCT was 521.81 ± 36.39 microns in cases compared to 520.99 ± 32.14 microns in controls. Thus, there was no significant difference between the two (p=0.163). 

**Table 3:** Comparison of Intraocular Pressure and corneal morphology parameters between cases and controls using Specular Microscopy

SN	Parameter	Cases (n=200)		Controls (n=200)		Statistical Significance	
		Mean	SD	Mean	SD	“t”	“p”
1.	IOP (mmHg)	15.47	2.02	15.60	2.04	0.616	0.538
2.	Number of cells counted (NUM)	226.86	68.84	261.84	39.43	6.375	< 0.001
3.	Cell density (CD) (/mm2)	2447.32	269.89	2570.70	221.22	5.000	< 0.001
4.	Average cell area (mm2)	413.97	50.19	392.16	30.33	4.942	< 0.001
5.	Standard deviation (SD)	167.05	77.91	146.42	30.33	3.490	0.001
6.	Coefficient of variance (%)	39.84	15.59	37.05	4.92	2.409	0.016
7.	Hexagonal cells (%)	45.03	6.71	47.50	6.88	3.637	< 0.001
8.	Central corneal thickness (CCT) µm	525.81	36.69	520.99	32.14	1.399	0.163

The mean ECC showed a substantial declining trend with increasing duration of diabetes (p=0.041), ECD being less in diabetics with > 2 years history of illness (p=0.013). The average cell area of those having a more prolonged duration of diabetes > 2 years was also significantly higher when compared to those with < 2 years of illness (p=0.050). 

Mean 6A was significantly lower in those having 1-10 years of diabetes as compared to those having < 1 and > 10 years. The mean CCT of those having > 5 years of diabetes was significantly lower compared to those having < 5 years of diabetes (p=0.004).

Mean cell density significantly declined with increasing fasting blood glucose levels (p=0.051). Mean hexagonal cells were lower in FBS < 400 mg/dl than those with FBS > 400 mg/dl (p=0.033), as shown in **Tables 4**-**6**. Increasing HbA1c significantly showed decreasing cell density and an increasing trend of average cell area (p < 0.001). When compared between NPDR and DR, cell density showed a decreasing trend, and average cells increased significantly (p < 0.05) (**Table 7**).

**Table 4:** Comparison of intraocular pressure, corneal morphology, and central corneal thickness in patients with different durations of diabetic history (n=200)

SN	Parameter	<1 Year (n=54)	1-2 Years (n=22)	2-5 Years (n=36)	5-10 Years (n=44)	> 10 Years (n=44)	Statistical significance (ANOVA)
1.	IOP (mmHg)	15.57 ± 1.79	15.82 ± 2.06	15.14 ± 2.19	15.48 ± 2.09	15.43 ± 2.11	F=0.440; p=0.779
2.	Number of cells counted (NUM)	248.67 ± 53.70	248.67 ± 53.70	222.58 ± 69.02	214.86 ± 72.11	211.66 ± 75.82	F=2.549; p=0.041
3.	Cell density (CD) (/mm2)	2526.04 ± 302.90	2545.64 ± 184.19	2412.64 ± 292.25	2402.43 ± 244.12	2374.82 ± 239.07	F=3.267; p=0.013
4.	Average cell area (mm2)	402.82 ± 62.38	394.96 ± 28.85	420.72 ± 53.43	420.34 ± 42.35	425.27 ± 42.24	F=2.422; p=0.050
5.	Standard deviation (SD)	152.22 ± 57.92	155.41 ± 52.98	170.58 ± 75.93	175.64 ± 89.81	179.59 ± 96.11	F=1.050; p=0.383
6.	Coefficient of variance (%)	37.46 ± 9.03	39.00 ± 11.65	40.50 ± 16.01	40.59 ± 17.33	41.88 ± 20.85	F=0.552; p=0.698
7.	Hexagonal cells (%)	47.65 ± 6.03	43.09 ± 6.75	42.97 ± 7.66	43.23 ± 6.74	46.25 ± 5.39	F=4.876; p=0.001
8.	Central corneal thickness (CCT) µm	525.98 ± 37.77	528.50 ± 25.87	544.81 ± 41.33	514.68 ± 30.20	519.84 ± 37.06	F=3.969; p=0.004

**Table 5:** Comparison of intraocular pressure, corneal morphology, and central corneal thickness in patients with different levels of fasting blood sugar (n=200)

SN	Parameter	FBS < 126 mg/dl (n=50)	FBS 126-200 mg/dl (n=88)	FBS 200-400 mg/dl (n=56)	FBS > 400 mg/dl (n=6)	Statistical significance (ANOVA)
1.	IOP (mmHg)	15.12 ± 1.85	15.90 ± 2.03	15.23 ± 2.11	14.33 ± 1.37	F=2.781; p=0.042
2.	Number of cells counted (NUM)	246.48 ± 64.84	226.35 ± 63.60	211.07 ± 68.24	218.00 ± 90.78	F=2.574; p=0.055
3.	Cell density (CD) (/mm2)	2666.42 ± 201.17	2448.69 ± 238.53	2275.60 ± 237.91	2204.17 ± 92.03	F=28.537; p<0.001
4.	Average cell area (mm2)	376.98 ± 27.11	413.07 ± 50.69	444.09 ± 44.87	454.33 ± 19.40	F=22.656; p<0.001
5.	Standard deviation (SD)	140.90 ± 36.50	173.67 ± 82.30	179.46 ± 92.95	172.00 ± 79.26	F=2.635; p=0.051
6.	Coefficient of variance (%)	37.24 ± 8.58	41.05 ± 15.68	40.45 ± 19.67	38.00 ± 17.67	F=0.692; p=0.558
7.	Hexagonal cells (%)	45.24 ± 6.50	45.00 ± 7.12	44.07 ± 6.10	52.50 ± 2.81	F=2.965; p=0.033
8.	Central corneal thickness (CCT) µm	531.12 ± 38.87	528.07 ± 36.32	517.68 ± 35.62	524.33 ± 27.31	F=1.388; p=0.248

**Table 6:** Comparison of intraocular pressure, corneal morphology, and central corneal thickness in patients with different levels of HbA1c (n=200)

SN	Parameter	HbA1 < 6.5% (n=64)	HbA1c 6.5-8.0% (n=80)	HbA1c 8.1-10.0% (n=38)	HbA1c > 10% (n=18)	Statistical significance (ANOVA)
1.	IOP (mmHg)	15.50 ± 2.04	15.30 ± 1.91	15.82 ± 2.12	15.39 ± 2.30	F=0.571; p=0.635
2.	Number of cells counted (NUM)	229.63 ± 73.16	235.18 ± 59.40	213.84 ± 63.51	207.50 ± 78.44	F=1.443; p=0.232
3.	Cell density (CD) (/mm2)	2548.58 ± 235.91	2458.91 ± 279.16	2337.79 ± 217.42	2267.00 ± 284.47	F=8.727; p<0.001
4.	Average cell area (mm2)	395.64 ± 36.47	412.83 ± 56.98	431.37 ± 40.02	447.50 ± 53.34	F=7.782; p<0.001
5.	Standard deviation (SD)	147.38 ± 55.36	168.84 ± 77.63	193.13 ± 95.92	174.00 ± 92.91	F=2.924; p=0.035
6.	Coefficient of variance (%)	37.19 ± 13.08	40.38 ± 14.90	43.58 ± 18.50	38.94 ± 19.36	F=1.406; p=0.242
7.	Hexagonal cells (%)	45.20 ± 7.00	44.75 ± 6.87	44.74 ± 5.89	46.22 ± 6.91	F=0.271; p=0.846
8.	Central corneal thickness (CCT) µm	525.33 ± 39.41	527.05 ± 35.72	529.74 ± 35.85	513.72 ± 32.83	F=0.828; p=0.480

**Table 7:** Comparison of Intraocular Pressure, Corneal Morphology and central corneal thickness in patients with different diabetic retinopathy status (n=200)

SN	Parameter	No DR (n=172)	NPDR (n=22)	PDR (n=6)	Statistical significance (ANOVA)
1.	IOP (mmHg)	15.48 ± 2.03	15.18 ± 2.02	16.17 ± 1.94	F=0.582; p=0.560
2.	Number of cells counted (NUM)	227.94 ± 69.68	226.36 ± 44.01	197.67 ± 50.61	F=0.593; p=0.554
3.	Cell density (CD) (/mm2)	2470.39 ± 261.42	2371.36 ± 223.25	2064.50 ± 364.56	F=8.071; p < 0.001
4.	Average cell area (mm2)	409.43 ± 44.55	425.36 ± 40.27	502.33 ± 122.15	F=11.708; p < 0.001
5.	Standard deviation (SD)	161.67 ± 70.40	187.55 ± 86.47	246.00 ± 174.27	F=4.396; p=0.014
6.	Coefficient of variance (%)	39.13 ± 14.69	43.46 ± 17.92	46.83 ± 28.55	F=1.380; p=0.254
7.	Hexagonal cells (%)	45.11 ± 6.71	44.56 ± 6.55	44.50 ± 8.26	F=0.086; p=0.918
8.	Central corneal thickness (CCT) µm	524.86 ± 35.93	528.50 ± 40.54	543.33 ± 45.67	F=0.800; p=0.451

## Discussion

Uncontrolled DM harms the cornea with 70% of diabetics resulting in complications like keratopathy. Although this topic has been extensively researched since the 1980s, there have been contradictory results regarding the direct effect of diabetes on corneal morphology. The difference in results shown by multiple studies can be attributable to the biased sampling of subjects. Some studies have taken both Type I and Type II DM cases as subjects, leading to a comparison between younger and older patients at the same time and not considering the effect of age-related degenerative changes in corneal morphology [**15**,**16**]. Few studies have enrolled diabetic subjects without evaluating the glycemic status of patients.

In this study, not only the duration of the disease but also the fasting blood glucose and HbA1c of each diabetic patient were included. This inclusion helped us understand the natural course of the disease and its effect on corneal morphology. The study highlighted that the increased duration of diabetes raised HbA1c, and poor glycemic control negatively affected corneal morphology.

A previous study reported a non-significant correlation between the duration of diabetes and ECD [**17**]. Modis et al. reported a significant correlation between HbA1c, plasma glucose level, and staging of DR with corneal parameters in Type-1 diabetics. Meanwhile, a non-significant relation was observed between the duration of diabetes, HbA1c, glucose level, and severity of DR, with corneal parameters also found in various studies [**2**,**18**,**19**]. 

Significantly lower ECD in diabetics versus healthy controls was reported in previous studies [**13**,**20**-**22**]. Few studies found lower CED values in Type-I diabetics compared to controls, whereas in Type-II diabetics the difference was not significant [**15**,**17**]. Another study reported ECD loss by 5% in Type-II diabetics and by 11% in Type-I diabetics when compared to healthy controls [**23**]. Moreover, our study showed a definite reduction in ECD and 6A in diabetics compared to non-diabetics, which few studies have shown. This difference is very well-attributable to the small sample size in other studies. Our sample size adequately strengthened the study’s power, and the age-matched groups avoided any confounding effect of age-related corneal degeneration. In contrast, few studies documented that diabetic subjects did not vary from non-diabetics in ECD [**12**,**22**].

This study further highlighted no correlation between CCT in the two groups, per preceding studies [**21**,**23**]. However, limited studies have reported a thicker cornea in diabetics [**24**-**27**]. This difference in agreement needs to be reinforced in the presence of biochemical factors, including glycemic controls, which may cause variations in swelling pressure and increased corneal thickness [**28**]. It is further pertinent to enforce that different studies have assessed various corneal parameters using different contact and non-contact equipment, which use automatic, semi-automatic, or manual methods. Hence, a comparison between studies needs to be more equitable. Our study used only one non-contact automatic specular microscope to evaluate all the corneal parameters, including CCT.

Moreover, our study had some limitations. The cross-sectional design of this study itself reduced its validity and further longitudinal study designs are needed. Follow-up studies may help further understand the pathophysiological effects. In addition, evaluating anatomical features of corneal endothelium may give indirect data about the functionality of endothelial cells, but conclusive evidence is needed.

## Conclusion

A significant association between reduction in corneal endothelial parameters has been observed in diabetics compared to non-diabetics. The parameters like endothelial cell density and hexagonality are also affected by increased duration of diabetes, poor glycemic control, and raised HbA1c levels. However, diabetes does not show any significant effect on CCT.

**Conflict of Interest Statement**

The authors state no conflict of interest.

**Informed Consent and Human and Animal Rights Statement**

Informed consent has been obtained from all individuals included in this study.

**Authorization for the use of human subjects**

Ethical approval: The research related to human use complies with all the relevant national regulations, and institutional policies, as per the tenets of the Helsinki Declaration, and has been approved by the review board of Military Hospital, Jalandhar, India (01/06/JUNE/MH/2023 dt 02 Dec 2023).

**Acknowledgments**

None.

**Sources of Funding**

None.

**Disclosures**

None.
